# A Clinical-Epidemiological Study on Beta-Blocker Poisonings Based on the Type of Drug Overdose

**DOI:** 10.1155/2023/1064955

**Published:** 2023-02-24

**Authors:** Nastaran Eizadi-Mood, Mahtab Adib, Arman Otroshi, Gholamali Dorooshi, Rokhsareh Meamar

**Affiliations:** Isfahan Clinical Toxicology Research Center, Isfahan University of Medical Sciences, Isfahan, Iran

## Abstract

**Background:**

Beta‐blockers carry a high risk of potentially causing fatal poisoning if overdosed. We aimed to assess the clinical and epidemiological characteristics of patients with beta-blocker poisoning.

**Methods:**

Patients were categorized based on the type of drug poisoning into propranolol, other beta-blockers, and the combination of beta-blocker groups, respectively. Demographic data, drug toxicity, and clinical, laboratory, and treatment information of different groups were compared.

**Results:**

During the study period, 5086 poisoned patients were hospitalized, of whom 255 (5.1%) had beta-blocker poisoning. Most patients were women (80.8%), married (50.6%), with a history of psychiatric disorders (36.5%), previous suicide attempts (34.6%), and intentional type of exposure (95.3%). The mean ± SD age of the patients was 28.94 ± 11.08 years. Propranolol toxicity was the most common among different beta-blockers (84.4%). There was a significant difference in age, occupation, education level, and history of psychiatric diseases with respect to the type of beta-blocker poisoning (*P* < 0.05). We observed changes in the consciousness level and need for endotracheal intubation only in the third group (combination of beta-blockers). Only 1 (0.4%) patient had a fatal outcome in toxicity with the combination of beta-blockers.

**Conclusion:**

Beta-blocker poisoning is not common in our poisoning referral center. Propranolol toxicity was most common among different beta-blockers. Although symptoms are not different among defined beta‐blocker groups, more severe symptoms are observed in the combination of the beta-blocker group. Only one patient had a fatal outcome in the toxicity with the combination of the beta-blocker group. Therefore, poisoning circumstances have to investigate thoroughly to screen coexposure with combined drugs.

## 1. Introduction

In general, poisoning is referred to as adverse effects that occur following the use of drugs or chemicals and is one of the important causes of morbidity and mortality [1]. Epidemiological studies have indicated that up to 75% of hospital admissions could be related to drug poisoning [2, 3]. One of the types of drug poisoning is beta-blocker poisoning. Beta-blockers are often nonspecific blockers with the aim of acting on the beta-1 receptor located in the heart and arteries. With beta-1 blocking, they reduce the number of heartbeats and lower blood pressure [4, 5]. Beta‐blocker overdosing carries a high risk of potentially causing fatal poisonings because of its strong therapeutic effect and rapid onset of action [6]. There is a belief that beta‐blocker overdosing poses a serious, life‐threatening risk to patients and is difficult to treat. In a large survey in the US poison centers in 2003, the case fatality rate of 7,415 cases treated in healthcare facilities was reported at 0.45% [7]. One study showed that cardiovascular mortality was 1.4% among 280 beta‐blocker exposures [8].

It seems that the most important factor involved in cardiovascular complications in beta-blocker poisoning is the concomitant use of drugs, especially calcium channel blockers, cyclic antidepressants, and neuroleptics [8–10]. Although, in the case of beta-blockers, if there are no symptoms up to 6 hours after oral administration, poisoning seems unlikely [11, 12]. The prognosis of beta-blocker poisoning is excellent, especially when it is along with prompt medical intervention. In the absence of such concurrency, exposure to a beta-blocker with membrane-stabilizing activity is associated with an increased risk of cardiovascular morbidity [8].

Evaluation of toxicity with beta-blockers indicated that propranolol was the only beta-blocker associated with seizures; of those who ingested more than 2 g of propranolol, two-thirds experienced a seizure [13]. Another study showed that different types of beta-blockers might have various levels of toxicity, while poisoning with propranolol and metoprolol was more frequent and associated with higher rates of cardiovascular complications [8].

Poisoning with these drugs has various consequences depending on the type of treatment intervention, the time interval between referral to health centers and the start of the action, and a series of factors depending on the patient. Therefore, in this study, we aimed to investigate the clinical and epidemiological data of patients with beta-blocker poisonings in the poisoning referral center in the central part of Iran. The prognosis of beta-blocker poisoning was assessed based on the type of drug toxicity by examining the patients' records.

## 2. Methods

This cross-sectional study was performed in 2021 at Khorshid Hospital, affiliated with Isfahan University of Medical Sciences. The records of all patients who were referred to our center in 2018 because of beta-blocker poisoning were reviewed. The study protocol was approved by the Research Committee of Isfahan University of Medical Sciences, and the Ethics Committee confirmed it (IR.MUI.MED.REC.1399.040).

**Figure 1:**
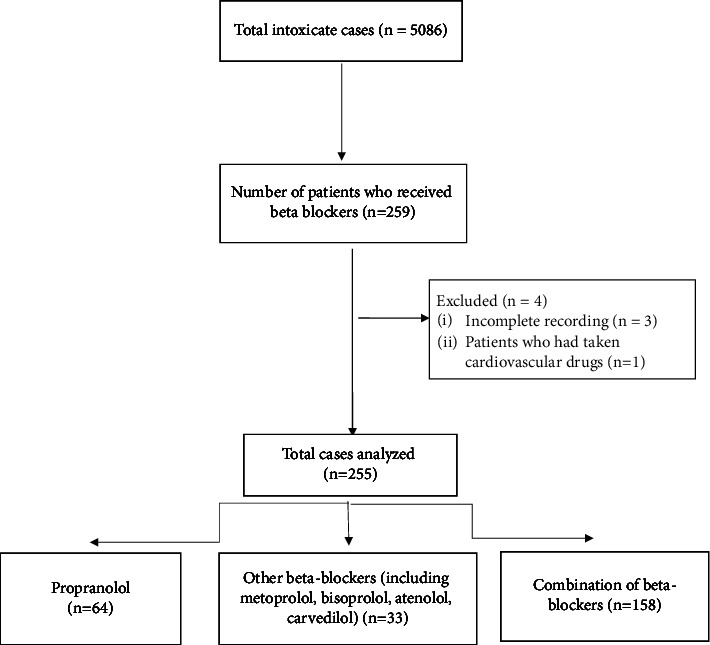
Flowchart.

The inclusion criteria were the age of more than 8 years, poisoning by beta-blockers, availability of medical records, and complete medical documents. Among 259 patients who were suspected of beta-blocker intoxication, 255 were included in the study. In multiple drug intakes, patients who had taken cardiovascular drugs (antihypertensive andantiarrhythmic) with beta-blockers were excluded from the study. Patients with a history of severe cardiac arrhythmia, renal and hepatic dysfunction, and those who left the hospital voluntarily or without permission while their follow-up was continuing were excluded. Patients were categorized into three groups according to the type of drug poisoning as propranolol, other beta-blockers (including metoprolol, bisoprolol, atenolol, and carvedilol), and the combination of beta-blockers, respectively (Figure 1).

The following information about poisoning was collected from the documents: personal characteristics (such as age, sex, marital status, level of education, and occupation), characteristics related to poisoning (type of drug, number of drug taken, and location of drug use), and mode of poisoning (intentional, accidental, and overdose), history of addiction and type of addiction (alcohol, cigarettes, opiates, or others), length of hospitalization, medical history related to psychiatric illness, and suicide history, as well as clinical findings in main organs including the central nervous system (CNS), heart, skin, eye (miosis or mydriasis), deep tendon reflex, palmar reflex, and vital signs (blood pressure, respiration rate, pulse rate, and body temperature) at baseline, laboratory data, treatments performed (receiving charcoal, atropine, glucose, calcium glucagon, dialysis) and other treatments, and treatment outcome (complete recovery or death). All poisonings registered in our medical center were collected after extracting the desired data and entering them into a computer file with a special format.

The obtained data were entered into the Statistical Package for Social Sciences (SPSS) version 24. Statistical analyzes were performed in two parts: descriptive and analytical. In the descriptive part, the reports were presented in the form of a percentage (number) for qualitative variables and an average (variance) for quantitative variables. In the analytical section, the relationship between age, sex, frequency of predictive factors, and outcome therapy was examined based on different outcomes by eliminating possible confounders using logistic regression. We used independent *t*-tests and repeated measure tests to compare data between different timelines and different groups. *P* < 0.05 was considered statistically significant.

## 3. Results

During the study period, 5086 patients were admitted to our medical center because of poisoning. Among 259 patients who were suspected of beta-blocker intoxication, 255 were included in the study. 255 (5.1%) patients were hospitalized because of beta-blocker poisoning. Among 255 patients who were performed in this study, only 1 (0.4%) had a fatal outcome. The mean ± SD age of the patients was 28.94 ± 11.08 years. Most patients (*n* = 206, 80.8%) were women, married (50.6%), housekeepers (53.3%), with a history of psychiatric diseases (36.5%), previous suicide (34.6%), and intentional ingestion (95.3%).

Data analysis showed that there were no significant correlations between the outcomes and the demographic characteristics, drug toxicity parameters, and medical history. Based on our analysis, the following characteristics were most prevalent among patients: married status, female sex, being a housewife or freelance worker, rural residence, hospital admission, intentional poisoning, drugs, nonaddicts, and consumption of cigarettes.

However, the pulse rate was significantly more normal among recovered patients (*P*=0.001), and the absence of intubation in the recovered group was strongly significant when compared with the fatality group (*P*=0.001).

**Figure 2:**
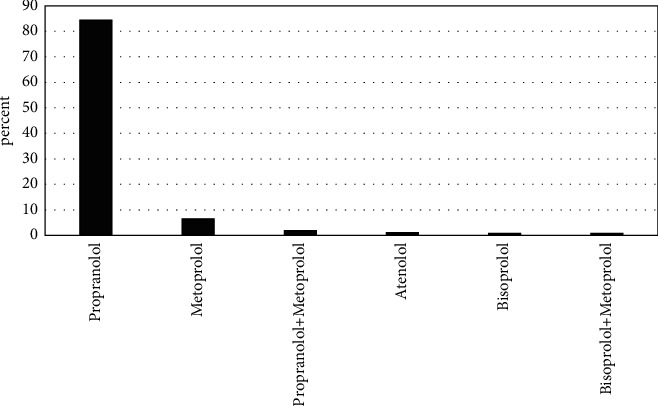
The frequency of beta-blocker poisoning.

Figure 2 shows the type of beta-blocker consumed. There was no significant relationship between the type of beta-blocker and the final outcome (*P*=1.00). Based on our results, propranolol was taken by 84.4% of the patients. Other types of beta-blockers were as follows: metoprolol (6.5%), atenolol (1.1%), bisoprolol (0.8%), propranolol and metoprolol (1.9%), and bisoprolol and metoprolol (0.8%). Beta-blockers were also taken orally by all patients, often at home.

Further analysis of demographic data between patients based on types of drug poisoning was shown at some points. We observed a significant correlation between age, occupation, education level, and history of psychiatric diseases based on the type of beta-blocker poisoning. A lower mean age was seen in patients poisoned with other beta-blockers (*P*=0.003). Most patients poisoned with propranolol were single, while married patients were mostly poisoned with a combination of beta-blockers (*P*=0.06). Beta-blocker overdose was mostly observed among housekeepers and students (*P*=0.015). Between the mentioned groups, we did not observe a significant difference based on addition and suicide history. A positive history of psychiatric diseases was more frequent among patients poisoned with a combination of beta-blockers (*P*=0.03, Table 1). 62% of the patients had taken a combination of beta-blockers.

In terms of CNS evaluation, we observed changes in the consciousness level in the combination of the beta-blocker toxicity group including two patients with coma, two patients with stupor, and four patients with restlessness. Endotracheal intubation was performed for six patients in the combination of the beta-blocker toxicity group (Table 1). Only 1 (0.4%) patient had a fatal outcome in toxicity with the combination of beta-blockers. All of the laboratory parameters did not differ between groups. In addition, charcoal therapy performed 25.5%, 12.1%, and 62.3% for propranolol, other beta-blockers, and the combination of beta-blocker groups, respectively.

## 4. Discussion

We aimed to evaluate beta-blocker toxicity and compare clinical and laboratory data among the types of beta-blockers. Beta-blocker poisoning is not common in our referral-poisoning center. In recent research, much attention has been given to the treatment procedure for beta-blocker toxicity, while few studies have evaluated its epidemiology and clinical manifestations. This might be due to the lower prevalence of suicide actions with beta-blockers in other regions and the higher rates of intentional beta-blocker toxicity in our society since they are prescribed for many reasons including problems such as headaches.

We observed that propranolol toxicity was most common among different beta-blockers followed by metoprolol. The indications are mainly used in the treatment of cardiovascular disease and migraine. In addition, propranolol is also used “off-label” to treat fear of social situations, panic disorder, and types of other anxiety disorders [14]. It could explain more prescriptions of propranolol and more toxicity of this drug when compared with others.

It was found that the patients of defined beta-blocker groups did not differ in terms of frequency and distribution of symptoms following exposure. In 2019, a study conducted by Lauterbach in Germany on 2967 cases of beta-blocker toxicity showed that there were significant differences between the occurrence and severity of symptoms among different types of beta-blockers [6].

In our study, there were more patients with bradycardia and coma status in the combination of the beta-blocker group. In addition, more intubations and deaths were also observed in this group. Only one case (0.4%) had a fatal outcome in the toxicity with the combination of the beta-blocker group. Lauterbach [6] recently indicated in a 10‐year retrospective, explorative analysis of the Mainz Poison Center/Germany database that all patients with beta-blocker toxicity recovered and only a potential fatality of coexposures with verapamil exposure occurred. It is compatible with our result that one fatality was observed in the group which was poisoned with the combination of beta-blockers. In another study in 2015, Menke et al. and colleagues evaluated the toxicity of cardiovascular xenobiotics in 10577 patients with a single exposure to beta-blockers. They showed that 9.6% of the patients had moderate-to-severe outcomes [15]. Indeed, in all the mentioned studies, the high recovery rate of patients was in line with our study in terms of a low mortality rate and good outcomes.

It has also been mentioned that cardiovascular complications such as bradycardia and hypotension are observed among most beta-blocker poisoning cases [6, 16]. In our study, symptoms were mainly cardiovascular with bradycardia only observed in 17 (6.6%) patients. A recent study evaluated beta-blocker poisoning and its required treatments. Their researchers found that beta-blocker poisoning is a serious clinical condition, mostly associated with cardiovascular manifestations, and immediate medical intervention is necessary. They also mentioned that there might be no significant differences between different types of drug poisoning and the clinical symptoms at the beginning of the process, while cardiovascular symptoms are the most important symptoms among them [17].

In our study, there were no statistically significant differences between groups in terms of mean age and sex which is in accordance with other studies [18].

A recent study in the US showed that almost 66% of beta-blocker poisoning was due to an accidental overdose or people using beta-blockers for noncardiac indications [19]. These data are not in line with the findings of our study which could be attributed to the higher prevalence of suicide actions in Iran compared with western countries [20, 21]. Based on our findings, patients with a combination of beta-blocker toxicities had a history of psychiatric diseases than the other groups. Based on previous evidence, the chances of multiple-drug toxicity are higher in patients with histories of psychiatric diseases [22].

Beta-blocker toxicity is often ascribed to the presence of membrane-stabilizing activity (MSA), a property of propranolol, labetalol, acebutolol, metoprolol, and pindolol. MSA was the only factor associated with cardiovascular toxicity [8]. Although hemodynamic compromise can result from beta-blockers without MSA, such instances appear to be much less common than with MSA [23, 24]. Since most of our patients were intoxicated with propranolol and metoprolol, it seems that the combination of beta-blocker toxicity with MSA properties might have induced more cardiac, CNS, and hemodynamic changes in our study. CNS depression was more frequently seen in combination with the beta-blocker group. Although lipophilic beta-blockers can cause CNS depression, combination with other medications including CNS depression can lower the level of consciousness in other beta-blockers as well.

Toxicity from beta-blocker exposure generally develops within 2 hours of ingestion [25]. A review of the literature [26] and a subsequent report [13] suggested that patients develop signs and symptoms of toxicity within 6 hours of ingestion. Our study substantiates these findings, showing that most of our patients with probable beta-blocker overdoses who remain asymptomatic and demonstrate no sign of hemodynamic instability for 6 hours after ingestion appear to be at little risk of subsequent deterioration. The mean time interval between poisoning and first treatment was 3.39 ± 4.8 hours, and this could explain the lower mortality in our cases due to the rapid therapeutic approach for these patients.

Activated charcoal was given to about 94% of the patients. Considering the rapid onset of action of beta-blockers after ingestion and impaired CNS status in a few patients with beta-blocker overdose, activated charcoal was used for almost all patients. However, this needs to be critically discussed for the potential risk of aspiration and should be further studied [6]. The limitation of our study was the small number of patients poisoned with single beta-blockers. Another limitation of our study was that beta-blocker doses were recorded according to patient reports, and the actual comparison between groups based on the blood concentration of drugs was not performed.

## 5. Conclusion

Beta-blocker poisoning is not common in our poisoning referral center. Propranolol toxicity was most common among different beta-blockers. Although symptoms have no difference among defined beta‐blocker groups, more severe symptoms are observed with the combination of beta-blocker groups. Only one patient had a fatal outcome in the toxicity with a combination of the beta-blocker group. Therefore, poisoning circumstances have to be thoroughly investigated to screen for coexposure to combined drugs.

## Figures and Tables

**Table 1:** Comparison of demographic, clinical symptoms, physical exam, and outcome based on the type of beta-blocker poisoning.

Variable		Propranolol *N* = 64	Other beta-blockers*N* = 33	Combination of beta-blockers*N* = 158	Total *N* = 255	*P* value
Age (year) (mean ± SD) Min-max 8–80		25.2 ± 8.7	16.93 ± 32.8	29.6 ± 10	28.94 ± 11.08	0.003

Sex (*N* (%))	Female	55 (26.7)	23 (11.2)	128 (62.1)	80.8%	0.15
	Male	9 (18.4)	10 (20.4)	30 (61.2)	19.2%	

Mean ± SD days of admission		1.56 ± 0.63	1.54 ± 0.56	1.75 ± 1.3	1.68 ± 1.1	0.36

Occupation (*N* (%))	Freelance worker	5 (11.9)	7 (16.7)	30 (71.4)	16.5%	0.01
	Employee	3 (30)	2 (20)	5 (50)	3.9%	
	Unemployed	0 (0)	1 (33.3)	2 (66.7)	1.2%	
	Housekeeper	30 (22.1)	16 (11.8)	90 (66.2)	53.3%	
	Student	25 (40.3)	6 (9.7)	31 (50)	24.3%	
	Child	1 (50)	1 (50)	0 (0)	0.8%	

Education level (*N* (%))	Illiterate	0 (0)	1 (100)	0 (0)	1.5%	0.05
	Secondary school	19 (48.7)	2 (5.1)	18 (46.2)	60%	
	Diploma	3 (18)	2 (12)	12 (70)	3.1%	
	License and higher	1 (12.5)	1 (12.5)	6 (75)	12.3%	

Type of poisoning (*N* (%))	Intentional	46 (25.8)	21 (11.8)	111 (62.4)	96.2%	0.39
	Accidental	1 (33.3)	1 (33.3)	1 (33.3)	1.6%	
	Overdose	2 (50)	0 (0)	2 (50)	2.2%	

Skin (*N* (%))	Normal	59 (24.6)	31 (12.9)	150 (62.5)	94.1%	0.47
	Cold and wet	0 (0)	1 (33.3)	2 (66.7)	1.2%	

Central nervous system (*N* (%))	Alert	47 (25.8)	25 (15.2)	93 (56.4)	64.7%	0.52
	Drowsiness	1 (20)	1 (20)	3 (60)	2%	
	Stupor	0 (0)	0 (0)	2 (100)	0.8%	
	Coma	0 (0)	0 (0)	2 (100)	0.8%	
	Restlessness	0 (0)	0 (0)	4 (100)	0.16%	

Heart auscultation (*N* (%))	Normal	54 (25.2)	28 (13.1)	132 (61.7)	84%	0.35
	Bradycardia	7 (41.2)	2 (11.8)	8 (47.1)	6.7%	

Systolic blood pressure (mmHg) (mean ± SD) Min-max		115.4 ± 15.37 79–150	122.6 ± 13.58 96–154	115.6 ± 17.37 12–171	12–171	0.07

Diastolic blood pressure (mean ± SD) Min-max		74.4 ± 11.1 47–100	78.5 ± 8.57 58–99	74.1 ± 13.08 40–112	40–112	0.16

Intubation (*N* (%))	Yes	0 (0)	0 (0)	6 (100)	2.4%	0.25

Outcome (*N* (%))	Recovery	64 (25.2)	33 (13)	157 (61.8)	97.7%	1.0
	Death	0 (0)	0 (100)	1 (0.6)	0.4%	

Time interval between poisoning and first therapeutic action (hour) (mean ± SD)		2.5 ± 1.77	4.5 ± 8.62	3.5 ± 4.46	3.39 ± 4.8	0.14

## Data Availability

The data used to support the findings of this study are available from the corresponding author upon request.
